# Nanoparticle modification in biological media: implications for oral nanomedicines

**DOI:** 10.1039/c9ra08403g

**Published:** 2019-12-06

**Authors:** Vishnaka Muraleetharan, Julia Mantaj, Magda Swedrowska, Driton Vllasaliu

**Affiliations:** Institute of Pharmaceutical Science, School of Cancer and Pharmaceutical Science, King's College London 150 Stamford Street London SE1 9NH UK Driton.vllasaliu@kcl.ac.uk

## Abstract

Nanomedicine has shown potential in enabling oral administration of poorly absorbable drugs, such as biologics. As part of the process related to optimisation of the safety and efficacy of nanomedicines, it is imperative that the interaction of nanoparticles with the biological systems – including the gut – is fully characterised. In this article, we provide an overview of the major mechanisms by which nanoparticles may transform upon introduction in biological media. Specifically, the phenomena of association, dissolution and biomolecule adsorption are discussed, together with factors which influence the occurrence of each phenomenon. The implications of these phenomena within the context of therapeutic action of nanomedicines, which includes reduced targeting efficiency, are also explored. Finally, we will comment on nanoparticle modification within the gut environment, including the currently available gastrointestinal models for the study of nano-bio interactions, with implications in the area of nanomedicines for oral administration.

## Abstract 2

Nanomedicines undergo transformation in biological media, which impacts biological effects. Such transformation in the gut environment has implications in use of nanomedicines for oral administration.

## Graphical abstract

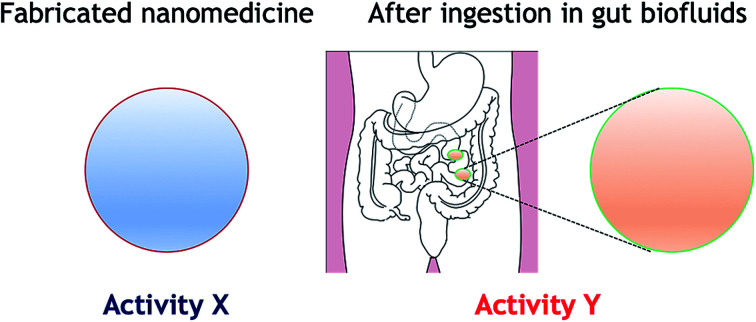

## 1. Introduction

The oral route of drug administration is favoured due to convenient administration (ingestion), which facilitates patient adherence to therapy. However, many drugs, including almost all biologics – the use of which has proliferated in recent years – are predominantly delivered *via* parenteral formulations because of poor oral bioavailability.^1^ This can be attributable to a number of factors, including drug degradation in the gastrointestinal (GI) tract and poor intestinal absorption.^2^ A number of drug delivery approaches are being researched to improve oral bioavailability of drugs such as biologics and poorly soluble drugs. One such approach makes use of drug carriers of nanoscale dimensions, *i.e.* nanomedicine. These nanosystems are usually based on polymeric nanoparticles that are designed to permeate the intestinal epithelium by targeting epithelial receptors which transport cargo from the luminal side to the basolateral (blood) side.^3,4^ Targeting of biological transport is thought to be essential as most particulates themselves show poor permeability across the GI mucosa.^5^

Nanoparticle biocorona formation, which arises as a result of interaction of nanoparticles with a range of biomolecules present in biological milieus (*e.g.* proteins, lipids, nucleic acids, metabolites), is a critical phenomenon that determines the biological fate (therapeutic activity or toxicity) of nanoparticles. The importance of the biomolecular corona formed around nanoparticles in biofluids in determining bioactivity (particle–cell interaction) has been demonstrated, for example following nanoparticle interaction with human plasma (as will be discussed later). However, characterisation of the biomolecular corona of nanoparticles in the gastrointestinal biofluids remains poorly understood.^6^ This knowledge is crucial since biofluid in the GI environments is complex and is likely to significantly alter the properties and colloidal stability of nanoparticles, and thus also their bioavailability and the biological responses they induce.^7^ The issue of nanoparticle modification in the gut and resulting implications for orally administered nanomedicines has been highlighted in a recent editorial by Berardi and Bombelli.^6^ This article aims to further increase the awareness of this (somewhat understudied) issue by providing a broader overview and discussion of nanoparticle behaviour in biological media and a focus on nanoparticle behaviours in the GI environment and consequences for oral nanomedicines.

## 2. The fate of nanoparticles in biological media and physiological implications

There are multiple mechanisms by which nanoparticles reduce their excess surface energy (to attain a more energetically stable state) on introduction to complex biological media.^8^ Following the classical Derjaguin–Landau–Verwey–Overbeek (DLVO) theory, nanoparticles may cluster into irregularly-shaped larger entities, namely soft agglomerates or dense aggregates.^9^ Alternatively, nanoparticles may dissolve, following the Noyes–Whitney relationships.^10^ Nanoparticles can additionally gain a corona of variable thickness around them, either by adsorbing biomolecules from the surroundings or by reacting with the surroundings (*e.g.* oxidation), which changes their initial identity.^9,11^

### 2.1. Association

The long-range thermodynamic forces between colloidal nanoparticles, governed by Brownian motion, result in interparticle collisions. As per the DLVO theory, nanoparticles may either associate (ir)reversibly or repel upon such contact, depending on their short-range thermodynamic interactions. Surface interactions are simplified by the theory, and the two forces considered to dominate short-range interparticle interactions are attractive van der Waal's (vdW) forces and repulsive electrostatic double layer (EDL) forces. Thus, the likelihood of nanoparticle association is approximately equal to the summation of these forces, which yields either a net attractive or net repulsive.

The colloidal stability of nanoparticles is influenced by both their inherent properties (*e.g.* shape, size and surface charge) and those of the medium in which they are suspended (*e.g.* pH and ionic strength). To exemplify, the larger a nanoparticle's net surface charge, the greater its colloidal stability. For nanoparticles bearing an amphoteric surface (*e.g.* metal hydroxides), surface charge is instead a function of the medium's pH. Under pH values close to their isoelectric point or point of zero charge, nanoparticles will have a near-neutral net surface charge. Interparticle repulsions due to the EDL will consequently be reduced, enhancing the effect of vdW attraction and encouraging nanoparticle association.^12^ Likewise, when subjected to low ionic strengths, a nanoparticle's EDL extends far from its surface, causing interparticle repulsions. Conversely, under high ionic strengths (as is often present in biofluids), the EDL experiences compression and neutralisation, causing nanoparticle association *via* a strengthened vdW force.^11^ By posing steric repulsion forces, surface coatings of synthetic polymer, polyelectrolytes or natural organic matter (NOM) may increase the colloidal stability of nanoparticles. Hydrophilic polymers, including polyethylene glycol (PEG) and polyvinylpyrrolidone (PVP), may enable further stabilisation *via* repulsive short-range hydration force.^12,13^

The association of nanoparticles in aqueous biological media, including cell culture media, may hinder their cellular targeting efficiency and reduce the representativity of experimental findings. Moreover, depending on their physicochemical properties and the cell type, nanoparticle association may skew their cellular uptake and toxicity profiles. For example, aggregated transferrin-capped gold nanoparticles experienced 25% reduced uptake by A549 and HeLa cells compared to their single, monodispersed counterparts. However, the largest aggregates underwent two-fold greater uptake by MDA-MB 435 cells. Nevertheless, no unique toxic responses were observed with the aggregation state.^14^

Conversely, Zook *et al.* identified a substantial decrease in hemolytic toxicity with an increase in size of silver nanoparticle agglomerates (from 43 nm to 1400 nm).^15^ Similarly, Tripathy *et al.* reported that, compared to small aggregates, large aggregates of zinc oxide nanoparticles exhibit drastically reduced potency with regards to evoking mitochondrial dysfunction, producing reactive oxygen species (ROS) and causing cellular apoptosis in RAW 264.7 murine macrophages. Interestingly, concentration-dependent aggregation was also identified here, wherein only small aggregates were formed at low concentrations of zinc oxide nanoparticles in phosphate buffer solution (PBS).^16^

Readers should note that although the association of pristine nanoparticles is possible in biomolecule-free media (*in vitro*), once in complex biological fluids, the adhesion of biomolecules on nanoparticles would change the surface characteristics of the latter. As a consequence of the new identity, nanoparticles could aggregate,^17^ or on the contrary, in some cases become more stable.^18^

### 2.2. Dissolution

Nanoparticle dissolution involves the movement of constituent molecules of a dissolving solid from its surface to the bulk solution, *via* a diffusion layer densely occupied by solvated entities, *e.g.* biomolecules, solute molecules and ions. Accordingly, the material's solubility in the medium and the surface-bulk concentration gradient are considered the major driving forces for this dynamic mechanism. As supported by the Noyes–Whitney equations, nanoparticles exhibit enhanced dissolution kinetics compared to bulk materials due to an immense surface-area-to-volume ratio, a reduced diffusion layer thickness and partially coordinated surface-residing atoms. Furthermore, with a greater proportion of these surface-residing atoms at edges and corners compared to at planar regions, ions and small clusters part from the surface more easily. Since dissolution can determine *in vivo* nanoparticle persistence, it should be considered upon the interpretation of their biological responses and toxicological profiles.^10,19,20^

Similar to nanoparticle association, dissolution is influenced by several factors, both intrinsic and extrinsic. Nanoparticles often demonstrate a kinetic size effect wherein the maximum concentration of dissolved species is observed instantaneously, after which the concentration decreases until saturation is attained. As identified by Schmidt and Vogelsberger for titanium dioxide nanoparticles, this phenomenon is most evident at larger nanoparticle concentrations.^21^ An enhanced kinetic size effect is generally associated with smaller nanoparticle sizes. For example, there is sufficient evidence in the literature illustrating an increase in the cytotoxicity of copper oxide when going from bulk/microscale material to smaller nanoparticles, by reason of increased solubility.^22,23^ Similarly, accelerated silver ion-release with a reduction in particle size, going from macroscale silver foil to nanoparticles of diameter 60 nm and 4.8 nm, has been reported.^24^ Conversely, no significant difference was observed between the dissolution rates of bulk and nanoparticulate zinc oxide in Osterhout's medium or a synthetic freshwater algal medium.^23,25^

In compliance with the Gibbs–Thomson effect, the dissolution tendency and equilibrium solubility of particles and surface structures increase with a reduced radius of positive curvature (*i.e.* convex); this is due to their energetic instability. In some instances, their equilibrium solubility may even surpass their saturation concentration, resulting in precipitation and/or Ostwald ripening. It can thus be deduced that nanoparticles bearing a reduced radius of negative curvature (*i.e.* concave) demonstrate better colloidal stability.^10^

As discussed above, the colloidal stability of nanostructures is often enhanced by the hindrance of interparticle association through surface coatings. However, the ligands of such stabilising agents may instead alter the dissolution kinetics of the nanoparticles they harbour.^19^ For instance, in water at 25 °C, PVP-stabilised silver nanoparticles have demonstrated a greater degree and rate of dissolution compared to citrate-stabilised silver nanoparticles. It was postulated that this was because the citrate layer served as a chemical barrier by reducing the departing silver ions.^26^ Contrastingly, Huynh and Chen observed that, over a 30 min duration in water, citrate-stabilised silver nanoparticles experienced greater dissolution than PVP-stabilised silver nanoparticles.^27^

As well as nanoparticle properties, the nature of the medium (*e.g.* pH and ionic strength) may influence nanoparticle dissolution through its effects on the above mentioned phenomenon, agglomeration and aggregation.^28^ The association of individual nanoparticles reduces their exposed surface area, thereby hindering ion release to the bulk medium and lowering the degree of dissolution. Additionally, the association of nanostructures reduces the number of surface sites which may be oxidised.^29^ For instance, the association of goethite nanorods at reduced pH values and/or increased ionic strengths was observed to quench, either largely or completely, their dissolution.^30^

Changes in the saturation concentration and dissolution kinetics of nanoparticles due to the availability of organic (*e.g.* NOM and polysaccharides) and/or inorganic (*e.g.* simulated biofluid and buffer) entities, are also documented in the literature. Dissolution is reportedly ‘catalysed’ by ionic and organic species capable of forming soluble complexes with the released ions. Conversely, the generation of less soluble complexes impedes dissolution.^10^ For instance, copper oxide nanoparticles have demonstrated near-complete dissolution in the presence of tryptone and yeast extract, which are rich in amino acids. However, no significant difference in dissolution was measured upon their exposure to sodium chloride.^22^ For silver nanoparticles, the addition of a small amount of chloride has been associated with a significant reduction in the rate of release of soluble species.^31^ Contrastingly, cysteine is reported to enhance the dissolution of silver nanoparticles.^32^

**Fig. 1:**
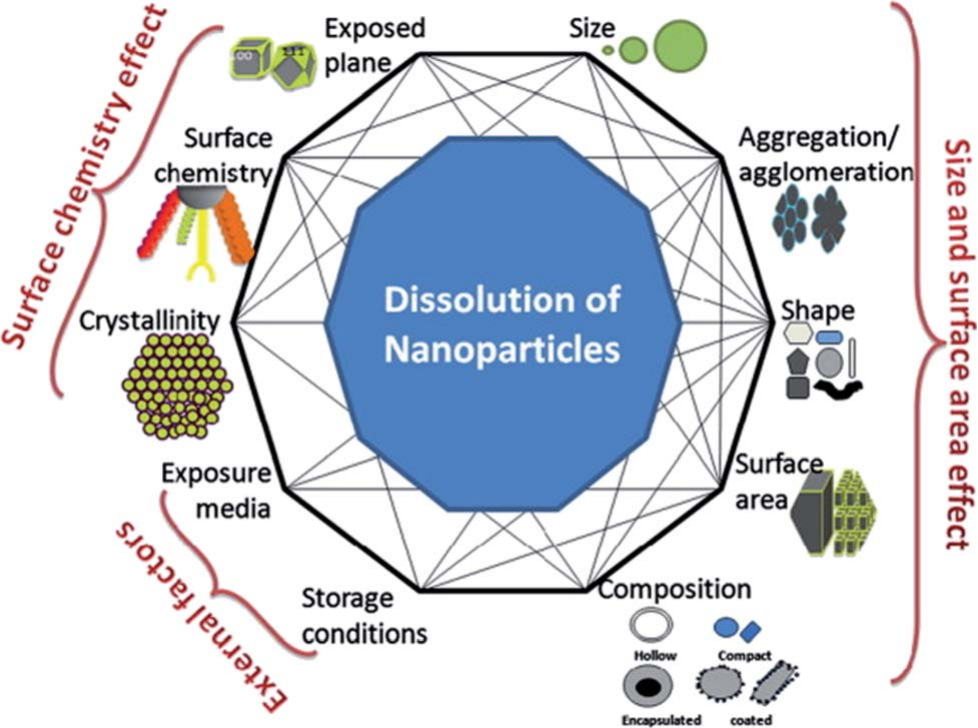
A simplistic representation showing the factors that can affect dissolution of nanoparticles and the possible interconnectivity among the factors, themselves. This figure has been reproduced from ref. 28 with permission from Elsevier.

The complex relationship between nanoparticle dissolution and the various intrinsic (*e.g.* nanoparticle composition, shape, surface area, surface chemistry, *etc.*) and extrinsic factors (*e.g.* media) is depicted in Fig. 1. Understanding these factors, facilitates the understanding and prediction of the fate and biological response of nanoparticles.

The immense contribution of dissolution to the cytotoxicity of nanoparticles comprising toxic constituent ions (*e.g.* zinc oxide), in comparison to that of nanoparticles which are themselves toxic (*e.g.* cerium dioxide), has been described in the literature.^33^ To exemplify, divalent and trivalent cations of copper and iron (respectively) may evoke site-specific DNA damage.^34^ Also, exposure to hexavalent and divalent cations of chromium and cadmium (respectively) has been shown to enhance hepatic lipid peroxidation.^35^ A greater proportion of copper nanoparticles (relative to copper carbonate salt) were reported to experience intestinal absorption, accumulate in the brain tissue and, at greater dietary doses, lead to hepatic damage in rat models.^36^ Furthermore, divalent silver and zinc cations are known to damage the bacterial membrane, thereby causing bacterial death.^37^

The employment of nanostructures (which may be considered a reservoir of cations) for medical applications therefore necessitates careful consideration. The extent of both the bactericidal and hemolytic activity of silver nanoparticles has correlated with the quantity of silver ions released.^15,38^ Likewise, oxidative stress and cytotoxicity by cadmium-containing quantum dots have been associated directly with the intracellular release of constituent divalent cadmium cations.^39^ However, while enhanced dissolution kinetics of nanoparticles has been employed in the case of drug nanocrystals to improve drug dissolution, oral nanomedicines are generally based on insoluble nanoparticulate carriers, designed to retain the drug until it reaches the target.

### 2.3. Biomolecule adsorption

On exposure to physiological milieus, nanoparticles may interact with a range of biomolecules including lipids, nucleic acids and metabolites. The adsorption of proteins to nanoparticle surface establish a protein corona has been given particular attention in the recent years, particularly within the context of nanomedicine targeting. Since many proteins are amphipathic, they have the potential to interact with almost any nanoparticle surface in their vicinity. Consequently, the interfacial interactions of nanoparticles with proteins are inevitable in such environments. In concordance, as expressed thermodynamically by Δ_ads_*G* < 0 (where Δ_ads_*G* symbolises the Gibbs free energy of adsorption), this mechanism of lowering the excess surface energy of nanoparticles is spontaneous.^40^

**Fig. 2:**
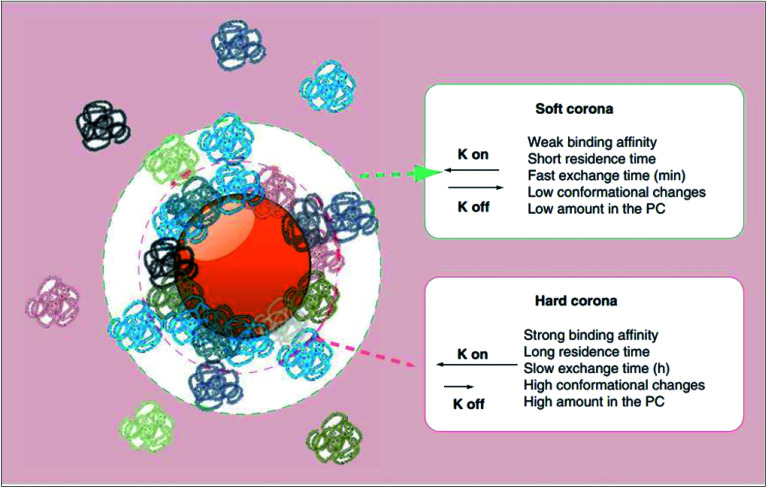
A schematic representation of the protein corona (PC) which branches into two layers, namely the hard and soft coronas. As listed, proteins which establish the hard corona exhibit higher affinity for the nanoparticle surface and thus have a longer surface residence time and slower exchange rate with proteins in the bulk medium than do proteins of the soft corona (as represented by a smaller rate constant (K off) in the order of hours as oppose to minutes). Hard corona proteins, of which there are more, also experience greater conformational changes than do soft corona proteins. This figure has been reproduced from ref. 11 with permission from Future Medicine.

The surface adsorption of serum proteins by particles (opsonisation) was first postulated by Wright and Douglas in 1903.^41^ Later in 1962, whilst assessing the surface adsorption of fibrinogen in blood plasma, Vroman noticed that the maximum surface concentration of fibrinogen was attained at an intermediate time point, suggestive of its substitution by other proteins with time.^42^ Described by the Vroman effect, corona formation is a dynamic and competitive process. The initial stages of adsorption are dominated by small (high mobility), abundant proteins. However, with time, some of these proteins are substituted by those which are larger (low mobility), less abundant and have greater affinity for the surface.^43^ An equilibrium is gradually attained, after which each protein has equal rates of association and dissociation, and further exchange causes no change in the corona's composition.^44^ As illustrated in Fig. 2, the protein corona branches into a ‘hard corona’ and ‘soft corona’; these denote a firmly bound, equilibrium state layer (wherein proteins exhibit high affinity for the nanoparticle surface), and a loosely bound layer (comprised of low-affinity proteins in rapid exchange with those in the medium), respectively.^45,46^

An intrinsic influencer of protein corona composition is nanoparticle size. For both citrate-stabilised and PVP-stabilised silver nanoparticles, proteomic analysis of the acquired corona revealed that 110 nm nanoparticles associated with almost 1.8 times more serum proteins than did 20 nm nanoparticles.^47^ The increased curvature of the smaller nanoparticles likely suppressed the adsorption of proteins, particularly those more bulky and rigid.^48^ Contrastingly, due to a size much smaller and bigger (respectively) than the most abundant serum proteins, neither 4 nm nor 40 nm gold nanoparticles acquired a complete protein corona.^49^ Concordantly, 30 nm gold nanoparticles were reported to adsorb 2.3 times more plasma proteins than 50 nm gold nanoparticles.^50^ This discrepancy suggests the effect of nanoparticle composition. Concerning protein conformation within the corona, 6 nm silica nanoparticles evoked a six-fold reduced modification in the secondary structure of human carbonic anhydrase 1 than did 15 nm silica nanoparticles.^51^ Similarly, increasing the size of silica nanoparticles from 4 nm to 15 nm substantially decreased the stability of adsorbed ribonuclease A due to increased protein unfolding.^52^

The association of nanoparticles in solution is known to both lower their exposed surface area for protein adsorption (which reduces the density of the corona) and induce surface rugosity (which evokes conformational changes in the adsorbed proteins).^53^ Regarding nanoparticle morphology, gold nanorods have been observed to undergo greater protein adsorption than gold nanospheres of equivalent size.^54^ Likewise, neutral nanoparticles have been reported to bind fewer proteins than their charged counterparts.^55^ Between positively-charged and negatively-charged nanoparticles, the former adsorb the greatest number of proteins.^56^ Moreover, employing gold nanoparticles bearing self-assembled monolayers of functionalised alkyl chains, it was proved that increasing surface hydrophobicity increases the corona's density. This is since protein adsorption is mediated (in part) by hydrophobic interactions.^57^

As demonstrated by Maiorano *et al.* utilising Dulbecco's Modified Eagle medium (DMEM) and Roswell Park Memorial Institute medium (RPMI), supplemented with Fetal Bovine Serum (FBS), biofluid composition is a prominent extrinsic influencer of corona formation. The two media (which vary in their salt, glucose and amino acid content) induced dissimilar adsorption patterns, with citrate-stabilised gold nanoparticles gaining a more abundant and stable corona in DMEM at all tested sizes.^58^ Consistently, an increase in plasma concentration (from 3% to 80%) corresponded to a notable increase in protein adsorption for polystyrene nanoparticles. For silica nanoparticles, increasing plasma concentration instead induced considerable changes in the protein pattern, illustrative of competitive adsorption by less-abundant, high-affinity proteins due to enhanced adhesion at higher plasma concentrations.^59^ Finally, nanoparticles which interact with proteins electrostatically have been reported to experience pH-dependent corona formation by reason of an altered protein binding affinity.^60^

**Fig. 3:**
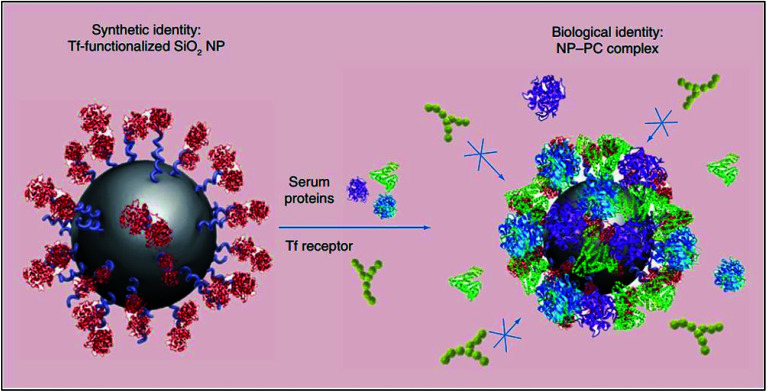
A schematic representation of the loss of transferrin (Tf)-mediated specificity by Tf-functionalised silicon dioxide nanoparticles due to the formation of a protein corona which impedes receptor-binding site interactions. This figure has been reproduced from ref. 11 with permission from Future Medicine.

The instantaneous adsorption of proteins by nanoparticles may alter their physicochemical attributes (including size and surface charge), thereby providing them with a new biological identity. Thus, it is not pristine nanoparticles, but those which are biomolecule-coated that largely govern physiological responses such as systemic circulation time, bioavailability, cellular uptake and cytotoxicity.^61^ For instance, the formation of a protein corona around transferrin-functionalised silicon dioxide nanoparticles was shown to eliminate their transferrin-mediated targeting capability due to masked receptor-binding sites (Fig. 3). In another study, the coating of gold nanoparticles with polyacrylic acid was shown to cause the unfolding of adsorbed fibrinogen which, consequently, evoked an inflammatory cascade.^11^

By contrast, serum proteins adsorbed to amine-functionalised polystyrene nanoparticles were reported to counterweight the nanoparticle surface charge, be retained upon their uptake (by both A549 and 1321N1 cells), and only experience degradation once trafficked to lysosomes. The harbouring of these positively charged nanoparticles averted their direct encounter with cell membranes, which would have otherwise induced cell death.^62^ Similarly, the generation of ROS (a measure of cytotoxicity) in human acute monocytic leukaemia (THP-1) cells by cobalt oxide nanoparticles was observed to be lower following incubation in complete cell culture medium for 48 h, than in PBS for the same duration.^63^

## 3. Nanoparticle modification in the gastrointestinal environment

### 3.1. The changing gastrointestinal environment

#### 3.1.1. Gastrointestinal fluid composition

GI fluids are highly complex and heterogeneous, with values of pH and ionic strength ranging from 1 to 8 and from 10 to >200 mM, respectively, inconsistent viscosity and the presence of various biomolecules (including bile salts, dietary food and liquid, digestive enzymes, lipids and lipolysis products). Furthermore, the GI environment is dynamic, evolving with both internal (*e.g.* gastric secretions) and external (*e.g.* food and/or liquid consumption) stimuli.^6^ Inter-subject (albeit more evident in the postprandial than in the preprandial state) and intra-subject variability (*e.g.* from regional specialisation) are also exhibited.^64,65^

Luminal fluid within the upper small intestine uniquely comprises chyme from the stomach, as well as secretions from the following compartments: liver (bile; bicarbonate, bile pigments, bile salts, cholesterol, phospholipids, and organic wastes), pancreas (bicarbonate, amylases, lipases and proteases including trypsin and chymotrypsin) and small intestinal wall (mineral ions; bicarbonate, chloride and sodium, and water).^66^ The digestion of silver nanoparticles with food compounds (carbohydrates, fatty acids and proteins) was reported to have a negligible impact on nanoparticle uptake by intestinal Caco-2 cells, whilst the digestion of silver nanoparticles in the absence of food compounds reduced cellular uptake by 60%.^67^ In another study, coatings of bovine serum albumin and casein were found to limit the adhesion of carboxylate-functionalised polystyrene nanoparticles (of 20 nm, 100 nm and 200 nm diameter) to Caco-2 cells. Conversely, exposure to meat extract caused no obvious impact on nanoparticle–cell interaction. Likewise, incubation in murine intestinal fluid increased the adherence of 20 nm and 100 nm nanoparticles to Caco-2 cells.^68^ Furthermore, treatment with *in vitro* digestive solution reportedly decreased the ROS generation potential of silicon dioxide and zinc oxide nanoparticles, albeit with no detectable effects on cytotoxicity.^69^

In addition to compartmentalisation, the composition of GI fluid is governed by mixing patterns, fluid absorption into the intestinal wall and transit through the intestinal tract. Several secretions of the small intestine are induced by food consumption, which gives rise to notable variation in GI fluid composition with prandial state. To exemplify, the consumption of a protein-rich meal (within the usual range of dietary intake) is followed by a surge in free and peptide amino acids within the intraluminal contents, originating from the exogeneous protein.^70^ Accordingly, the influence of protein-, fat- and carbohydrate-rich meals, as well as high-calorie meals, on drug absorption have been postulated.^71^ Thus ultimately, the prediction of nanoparticle transformation within the GI environment necessitates the consideration of regional and prandial effects on GI fluid composition, and thereby biomolecule corona formation.

#### 3.1.2. Changes in mechanical forces and pH

Numerous contractile motions occur within the bowel wall during normal gut function, both in the circular and longitudinal muscle layers. Peristaltic contractions of the circular and longitudinal muscles demonstrate high synchronisation.^72^ Within the stomach, peristaltic forces often range between 5 and 20 mmHg, however pressures of up to 150 mmHg have been measured occasionally.^73^ Ring and segmental contractions of the small bowel (intestine) serve predominantly to exert a propulsive force. These follow frequencies of 7 to 20 per minute, wherein the faster frequencies are characteristic of the duodenum, and the slower frequencies are found moving towards the distal ileum.^72^ However, despite considerable variability in the strength of such mechanical forces, research concerning the effect of intestinal contractions on the primary particle size distribution and aggregation state of nanoparticles appears to be lacking.^73^

The preprandial stomach is characterised by acidic pH values of 1.0 to 2.5, whilst average pH values of 6.6 and 7.5 are found in the proximal small intestine and terminal ileum, respectively.^74^ By contrast, the ingestion of food or liquid may cause a steep rise in gastric pH, up to values of 6.0 or higher depending on the composition and volume of the ingested matter.^75^ Similarly, the pH of the gastric mucus layer increases from approximately 1.0 in the lumen, to near-neutral values at the epithelial cell surface.^76^ A rise in pH towards the point-of-zero-charge of titanium dioxide nanoparticles has shown to increase the diameter and decrease the mobility of nanoparticle aggregates. As supported by the DLVO theory, this is due to the suppression of their surface ionisation which weakens interparticle EDL repulsions, thereby promoting nanoparticle aggregation.^77,78^ Thus, the inconsistency of GI pH must also be considered as a potential influencer of the therapeutic efficacy of nanoparticles and nano-scale drug delivery systems, through its modification of nanoparticle surface properties.

### 3.2. Gastrointestinal models for the study of nanoparticle behaviour

Major impediments to the characterisation of interactions between nanoparticles and the biological environment within the gut, which may explain the knowledge gap in this area to date, stem from the unavailability of reliable models of the GIT, in addition to analytical difficulties presented by the complex mixture of biomolecules (*e.g.* lipids, proteins and surfactants) within GI fluids. *In vivo* studies obviously enable a more reliable evaluation of nanomedicines, including establishing their toxicological profile, therapeutic response, drug–drug and drug–food interactions (and subsequent adverse effects), which are otherwise unidentifiable *in vitro*.^79^ However, it is imperative to select an animal model which best simulates the relevant human system. For example, rats are commonly employed to predict the human intestinal permeation of conventional chemicals. However, as a prerequisite, they must undergo surgery and anaesthesia, which may cause abnormal GI transit times. As a result of ethical considerations, costs, and inter-species variability, there is considerable interest in using representative *in vitro* models of the GI biofluids and GIT tissue for the study of nanoparticle behaviour post-ingestion/administration.

#### 3.2.1. Simulated intestinal fluids (biorelevant media)

Oral nanomedicines for systemic effect are assumed to be administered *via* a suitable oral dosage form (*e.g.* enteric capsule) that remains intact in the stomach and releases its content in the small intestine as the primary GI region for absorption. Human intestinal fluids (HIF) may be considered the ‘gold standard’ for the comprehension of nanoparticle behaviour following oral administration. Despite this, considerable research has been dedicated to the development of representative conditions (including pH, osmolality and composition). Concordantly, existing studies concerning the GI environment have predominantly utilised simulated intestinal fluids (SIF). This is because the isolation of HIF in volumes large enough for meaningful drug development studies is unfeasible. The composition of HIF is also variable, with the donor's prandial state (as mentioned previously) and the extraction procedure being major influencers.^80^ For a review on intestinal physiology, including gastrointestinal fluid, within the context of drug delivery, the reader is directed elsewhere.^81^

Fasted and fed state SIF (FaSSIF-V2 and FeSSIF-V2, respectively) are highly simplistic models of intraluminal composition. As such, they are deprived of vital intraluminal components which may render *in vitro* findings unreliable. For example, whilst HIF harbour a variety of bile salts, FaSSIF-V2 and FeSSIF-V2 comprise pure sodium taurocholate only.^82^ FeSSIF-V2 additionally fails to replicate the intricate ultrastructure of postprandial HIF (which includes mixed micelles and vesicles), likely due to the absence of lipids and lipolysis products. Furthermore, the movement and enzymatic degradation of food (which impact colloid formation) within the GIT are unaccounted for.^64,83^

The addition of pancreatin (and calcium chloride) is one approach to improve the complexity of FeSSIF-V2.^84^ The substitution of sodium taurocholate with crude bile salt extracts (in both FaSSIF-V2 and FeSSIF-V2) has also proven to better predict *in vivo* conditions. However, to ensure the reproducibility of experimental results, it is often necessary to standardise the quantity of bile salt added to crude extract batches.^85^

#### 3.2.2. *In vitro* cell models

Cell culture models enable the high-throughput and reproducible screening of mechanisms by which chemicals and nanomedicines may undergo transepithelial permeation. Nonetheless, whilst primary epithelial cells (extracted freshly from the GIT) may be considered the ideal system, their employment is limited by their short lifespan in culture and poor ability to form organised monolayers. Accordingly, *in vitro* studies on the intestinal absorption of chemicals and nanomedicines generally utilise immortalised cell lines which can yield adherent and GIT-representative monolayers.^86^ Various cell lines (each reflecting a different region of the GIT) are available, amongst which the intestinal Caco-2 cell line is highly popular. This is because Caco-2 cells model enterocytes as the most abundant small intestinal epithelial cell type.^87^ Also, when grown to confluence on semi-permeable inserts, Caco-2 cells differentiate spontaneously into a polarised monolayer bearing apical brush border microvilli, intercellular tight junctions and a variety of biorelevant metabolic enzymes.^88^ The Caco-2 model has been employed widely for the study or nanomedicines for oral delivery.^3,89,90^

However, the Caco-2 transwell system accounts for neither the constant fluid flow nor the fluid shear stress tolerated by epithelial cells *in vivo*, thereby skewing the predicted bioavailability of chemicals and/or nanomedicines.^91,92^ Caco-2 cell monolayers additionally lack a mucus coating, which serves to (i) control the intestinal absorption of matter and (ii) shield the epithelium from harmful intraluminal contents *in vivo*.^89^ Expanding on the latter, bile salts are considerably toxic (irritant) to epithelial cells despite being a major constituent of intestinal media. Thus, the potentially detrimental impact of SIF on the Caco-2 intestinal barrier model (which may otherwise be a useful strategy to model both the intestinal luminal fluid and epithelium) may cause the overestimation of transepithelial permeation.^93^ One strategy to omit this is to instead employ co-culture models (*i.e.* Caco-2 cells with mucus-secreting HT29-MTX cells).^94^

#### 3.2.3. Dynamic gastric model

Originally developed by the Institute of Food Research (Norwich; UK), the dynamic gastric model (DGM) aims to simulate the intricate physical mixing and emptying conditions of the human gastric system. By means of a fixed outer cylinder and mobile inner cylinder (between which foods are mechanically crushed), the effect of various food matrices on the *in vivo* processing of ingested nanoparticles (*e.g.* protein corona composition) may be assessed. However, the type and strength of the forces applied by the DGM often differ from those encountered during peristaltic contractions. Furthermore, DGM lacks the representation of biological components of the GIT (*e.g.* mucus/mucins), which may play a crucial role in nanoparticle transformation. Nevertheless, DGM may have a role in the study of nanomedicines for oral delivery although further research concerning the biorelevance of DGM-based protocols for the pharmaceutical assessment of these systems is required.^92,95^

## 4. Conclusions

Nanoscale drug delivery systems have been proposed and are being developed to improve oral drug bioavailability. However, detailed characterisation of the interactions between these nanosystems and the biological environment within the gut is currently lacking, with existing literature on the interactions and behaviour changes of nanoparticles in physiological environments focusing on the blood. Only limited studies have assessed the modification of nanoparticles on exposure to the GI tract conditions (*e.g.* digestion) and these point to significant modification of nanosystems in the GI environment, impacting their bioactivity (including cell uptake and toxicity). More research is needed in this area, particularly considering the complexity of the GI tract and the different factors that could influence nanoparticle behaviour, including the changing gastrointestinal fluid composition and mechanical forces. Importantly, study of nanoparticle behaviours in the GI environment has to precede and inform research into the design and development of oral nanomedicines. Otherwise, current studies focusing on formulation of nanoparticles for oral delivery, particularly those conducted *in vitro* and utilising simple and non-representative models (*e.g.* media), may be of limited value.

## Conflicts of interest

There are no conflicts to declare.

## Supplementary Material
